# Association Between Intraventricular Alteplase Use and Parenchymal Hematoma Volume in Patients With Spontaneous Intracerebral Hemorrhage and Intraventricular Hemorrhage

**DOI:** 10.1001/jamanetworkopen.2021.35773

**Published:** 2021-12-03

**Authors:** Jens Witsch, David J. Roh, Radhika Avadhani, Alexander E. Merkler, Hooman Kamel, Issam Awad, Daniel F. Hanley, Wendy C. Ziai, Santosh B. Murthy

**Affiliations:** 1 Clinical and Translational Neuroscience Unit and Department of Neurology, Feil Family Brain and Mind Research Institute, Weill Cornell Medicine, New York, New York; 2 Department of Neurology, University of Pennsylvania School of Medicine, Philadelphia; 3 Department of Neurology, Vagelos College of Physicians and Surgeons, Columbia University, New York, New York; 4 Brain Injury Outcomes Division, Johns Hopkins University School of Medicine, Baltimore, Maryland; 5 Department of Neurological Surgery, University of Chicago School of Medicine, Chicago, Illinois; 6 Division of Neurosciences Critical Care, Department of Neurology, Johns Hopkins University School of Medicine, Baltimore, Maryland

## Abstract

**Importance:**

Intraventricular thrombolysis reduces intraventricular hemorrhage (IVH) volume in patients with spontaneous intracerebral hemorrhage (ICH), but it is unclear if a similar association with parenchymal ICH volume exists.

**Objective:**

To evaluate the association between intraventricular alteplase use and ICH volume as well as the association between a change in parenchymal ICH volume and long-term functional outcomes.

**Design, Setting, and Participants:**

This cohort study was a post hoc exploratory analysis of data from the Clot Lysis: Evaluating Accelerated Resolution of Intraventricular Hemorrhage phase 3 randomized clinical trial with blinded outcome assessments. Between September 1, 2009, and January 31, 2015, patients with ICH and IVH were randomized to receive either intraventricular alteplase or normal saline via an external ventricular drain. Participants with primary IVH were excluded. Data analyses were performed between January 1 and June 30, 2021.

**Exposure:**

Randomization to receive intraventricular alteplase.

**Main Outcomes and Measures:**

The primary outcome was the change in parenchymal ICH volume between the hematoma stability and end-of-treatment computed tomography scans. Secondary outcomes were a modified Rankin Scale score higher than 3 and mortality, both of which were assessed at 6 months. The association between alteplase and change in parenchymal ICH volume was assessed using multiple linear regression, whereas the associations between change in parenchymal ICH volume and 6-month outcomes were assessed using multiple logistic regression. Prespecified subgroup analyses were performed for baseline IVH volume, admission ICH volume, and ICH location.

**Results:**

A total of 454 patients (254 men [55.9%]; mean [SD] age, 59 [11] years) were included in the study. Of these patients, 230 (50.7%) were randomized to receive alteplase and 224 (49.3%) to receive normal saline. The alteplase group had a greater mean (SD) reduction in parenchymal ICH volume compared with the saline group (1.8 [0.2] mL vs 0.4 [0.1] mL; *P* < .001). In the primary analysis, alteplase use was associated with a change in the parenchymal ICH volume in the unadjusted analysis per 1-mL change (β, 1.37; 95% CI, 0.92-1.81; *P* < .001) and in multivariable linear regression analysis that was adjusted for demographic characteristics, stability ICH and IVH volumes, ICH location, and time to first dose of study drug per 1-mL change (β, 1.20; 95% CI, 0.79-1.62; *P* < .001). In the secondary analyses, no association was found between change in parenchymal ICH volume and poor outcome (odds ratio [OR], 0.97; 95% CI 0.87-1.10; *P* = .64) or mortality (OR, 0.97; 95% CI 0.99-1.08; *P* = .59). Similar results were observed in the subgroup analyses.

**Conclusions and Relevance:**

This study found that intraventricular alteplase use in patients with a large IVH was associated with a small reduction in parenchymal ICH volume, but this association did not translate into improved functional outcomes or mortality. Intraventricular thrombolysis should be examined in patients with moderate to large ICH with IVH, especially in a thalamic location.

## Key Points

**Question:**

Is intraventricular thrombolysis in patients with spontaneous intracerebral hemorrhage (ICH) and intraventricular hemorrhage (IVH) associated with reduced parenchymal ICH volume?

**Findings:**

In this cohort study of 454 patients with ICH and IVH who were enrolled in a randomized clinical trial, intraventricular alteplase use was associated with a small reduction in the parenchymal ICH volume. However, no association was found between a change in parenchymal ICH volume and death or major disability.

**Meaning:**

Findings of this study suggest that further exploration of the role of intraventricular thrombolysis in reducing parenchymal ICH volumes and potentially improving outcomes is warranted in patients with a moderate to large ICH with IVH and those with a thalamic ICH.

## Teaser

This cohort study evaluates the mortality and functional outcome in patients with spontaneous intracerebral hemorrhage who received intraventricular alteplase vs those who received saline.

## Introduction

About one-third of patients with spontaneous intracerebral hemorrhage (ICH) die within 1 month of the event, and almost half of patients die during the first year after the index hemorrhage.^1^ Despite comprising only 10% to 15% of all stroke subtypes, ICH is responsible for more than two-thirds of overall stroke mortality.^2^ Among the factors associated with poor ICH outcomes, the volume of intracranial blood has been identified as one of the most important; other variables include the initial parenchymal ICH volume at the time of ICH diagnosis, subsequent hematoma expansion, and intraventricular hemorrhage (IVH).^3,4^ Removal of both parenchymal hematoma and IVH has been tested as a treatment target in large randomized clinical trials, including the Clot Lysis: Evaluating Accelerated Resolution of Intraventricular Hemorrhage phase 3 (CLEAR III) trial; however, these trials found decreased mortality but no improvement in functional outcome.^5,6,7,8^

A recent single-center retrospective pilot study in patients with ICH and IVH highlighted that alteplase administered via an external ventriculostomy might be associated with reduced parenchymal ICH volume in addition to its known ability to lower intraventricular blood volume.^9^ Although hypothesis generating, the pilot study lacked power to assess any potential association between diminished parenchymal ICH volume and disability. An association between intraventricular alteplase use and reduction in parenchymal ICH volume could be a novel therapeutic intervention particularly in the absence of IVH.

Therefore, we sought to evaluate the association between intraventricular alteplase use and ICH volume and subsequently examine the association between a change in parenchymal ICH volume and long-term functional outcomes. To test these hypotheses, we leveraged data from a cohort of patients with a large IVH who underwent serial neuroimaging and a robust follow-up as part of a large randomized clinical trial.

## Methods

This post hoc cohort study was approved by the institutional review board of Weill Cornell Medicine. Written informed consent that was obtained from participants in the CLEAR III trial extends to the present study. We followed the Strengthening the Reporting of Observational Studies in Epidemiology (STROBE) reporting guideline.^10^ The CLEAR III trial protocol was approved by the institutional review board at each participating site, and written informed consent was obtained from all participants or their legal representatives or surrogates when applicable.

### Design and Study Population

We conducted a post hoc exploratory analysis of data that were prospectively collected in the CLEAR III trial, a multicenter, double-blind, placebo-controlled randomized clinical trial that examined whether a pragmatically used external ventricular drain (EVD) with intraventricular alteplase irrigation of the ventricular system improved outcomes by removing IVH and controlling intracranial pressure vs EVD with saline irrigation.^7^ In the CLEAR III trial, the main inclusion criteria were (1) age between 18 and 80 years; (2) spontaneous ICH with parenchymal ICH volume less than 30 mL; (3) obstruction of the third or fourth ventricles; (4) presentation within 24 hours of symptom onset; (5) stability of ICH, IVH, and any EVD tract hemorrhage within 72 hours of the diagnostic noncontrast computed tomography (CT) scan; and (6) baseline modified Rankin Scale score lower than 2. Randomization, which was conducted between September 1, 2009, and January 31, 2015, had to be done within 72 hours from time of the diagnostic CT scan. However, a patient was eligible for randomization only if the clot size was stabilized between 2 sequential CT scans at least 12 hours apart. This measure was defined as hematoma stability, and the resultant ICH or IVH volumes were termed *stability volumes*.^11^ Patients were randomized to receive either up to 12 doses of alteplase or 0.9% saline every 8 hours through the EVD until the third and fourth ventricles were radiographically open. Details of the CLEAR III trial methods and results have been published elsewhere.^7^

For the current cohort study, we excluded all patients with primary IVH (ie, without a parenchymal hematoma component on the initial CT scan). Patient demographics and comorbidities were recorded at the time of trial enrollment. Baseline characteristics included age, sex, comorbidities, and admission severity variables, such as Glasgow Coma Scale score and National Institutes of Health Stroke Scale score.

### Neuroimaging

In the CLEAR III trial, noncontrast CT scans were obtained on admission, at 12-hour intervals until stability, and once daily after randomization, until after the last dose of the study agent to achieve 80% removal of IVH volume. Parenchymal ICH volumes were calculated using semiautomated planimetry and were read centrally by radiologists and neurologists who were blinded to treatments and outcomes. The ICH and IVH volumes were calculated at diagnosis, at stability (ie, no further evidence of new bleeding from any site), and at the end of treatment (ie, 24 hours after the last dose of the study agent). Other radiologic parameters ascertained were ICH location and perihematomal edema volumes. Lobar ICH was defined as the selective involvement of cerebral cortex, underlying white matter, or both, whereas deep ICH was defined as the selective involvement of thalami, basal ganglia, or both.^12^

### Measurements

In this cohort study, the exposure was intraventricular alteplase administration. The primary outcome was a change in the parenchymal ICH volume from stability to the end of treatment. The secondary outcomes were a composite of major disability or death (defined as a modified Rankin Scale score >3) and mortality, both of which were assessed at 6 months.

### Statistical Analysis

We reported data as counts (percentages) or medians (IQRs), unless specified otherwise. To test for intergroup differences, we used the Mann-Whitney and unpaired, 2-tailed *t* tests for continuous variables (depending on the normality of distribution) and the χ^2^ test for categorical variables. Change in ICH volume was measured as the difference between stability ICH volume and end-of-treatment ICH volume and was treated as a continuous variable.

We conducted 2 different analyses. First, we assessed the association between alteplase use and change in parenchymal ICH volume using linear regression. We ensured that the assumptions for linear regression, including linearity, homoscedasticity, and multicollinearity were met. Multicollinearity was identified using a variance inflation factor value higher than 4. Because we excluded few patients from the CLEAR III trial in the present study, we assumed that the original trial randomization would still apply. Therefore, we performed an unadjusted linear regression analysis first, followed by a multivariable linear regression. Covariates for the multivariable linear regression model were identified by *P* < .20 in the bivariate analysis. In the model, we also used universal confounders, such as age, sex, and race and ethnicity (which were investigator-reported in the CLEAR III trial and were categorized as follows: Black, Hispanic/Latino, White, or Other [Asian, Native American, Native Hawaiian, or other Pacific Islander]), regardless of their significance in the bivariate analyses. The model was adjusted for age, sex, race and ethnicity, stability ICH volume, ICH location, stability IVH volume, and time from ictus to first dose of study agent. Although linear regression has been shown to be robust for model assumptions in randomized clinical trials,^13^ we performed model diagnostics and found that heteroscedasticity was present. We tried to address heteroscedasticity by using robust SEs and weighted least squares regression.

Second, we examined the association between change in ICH volume and 6-month functional outcomes using multiple logistic regression. The models were adjusted for known factors of poor outcome, such as age, admission ICH volume, presence of IVH, hematoma location, and admission Glasgow Coma Scale score.

In addition, we performed prespecified analyses with stratification by baseline IVH volume (≥20 mL vs <20 mL), admission ICH volume (≥10 mL vs <10 mL), and ICH location (thalamic vs nonthalamic). The rationale for these analyses was to evaluate the effect modification by the baseline ICH severity factors.

Statistical analyses were performed using Stata, release 16 (StataCorp LLC). The threshold for statistical significance was a 2-sided *P* < .05. Data analyses were performed between January 1 and June 30, 2021.

## Results

Among the 500 patients who were enrolled in the CLEAR III trial, 46 with primary IVH were excluded. The final analytical cohort comprised 454 patients, of whom 254 were men (55.9%) and 200 were women (44.1%) with a mean (SD) age of 59 (11) years.

A total of 230 patients (50.7%) received intraventricular alteplase (treatment group), whereas 224 patients (49.3%) received saline (control group). Compared with patients in the control group, those in the treatment group had a lower proportion of hypertension (67.4% [n = 155] vs 79.9% [n = 179]; *P* = .002), whereas the prevalence of other cardiovascular comorbidities was similar across the 2 groups (Table 1). No observable differences in the ICH characteristics were found, with the exception of median (IQR) end-of-treatment IVH volume, which was lower in the alteplase group than in the saline group (5.6 [1.9-13.0] mL vs 9.9 [5.6-20.8] mL; *P* < .001). The median (IQR) end-of-treatment parenchymal ICH volume was numerically but not statistically significantly smaller in the alteplase group compared with the control group (7.3 [2.5-14.4] mL vs 8.6 [3.1-14.1] mL; *P* = .32). The alteplase group had a greater mean (SD) reduction in parenchymal ICH volume compared with the saline group (1.8 [0.2] mL vs 0.4 [0.1] mL; *P* < .001) (Figure).

**Table 1.** Baseline Characteristics of Patients With Intracerebral Hemorrhage

Characteristic	No. (%)		*P* value^a^
	Alteplase group (n = 230)	Saline group (n = 224)	
Age, mean (SD), y	57.9 (11.5)	59.2 (10.8)	.33
Sex			
Female	96 (41.7)	104 (46.4)	.31
Male	134 (58.3)	120 (53.6)	
Race and ethnicity^b^			
Black	86 (37.4)	68 (30.3)	.42
Hispanic/Latino	28 (12.2)	30 (13.4)	
White	109 (47.4)	116 (51.8)	
Other^c^	7 (3.0)	10 (4.5)	
Hypertension	155 (67.4)	179 (79.9)	.002
Hyperlipidemia	221 (96.1)	219 (97.8)	.30
Tobacco use	67 (29.1)	48 (21.4)	.06
Previous anticoagulant use	16 (6.9)	24 (10.7)	.16
Previous antiplatelet use	51 (22.2)	65 (29.0)	.10
Clinical severity factor			
Glasgow Coma Scale score at screening, points^d^	10 (7-13)	9 (7-12)	.57
ICH location			
Lobar	23 (10.0)	30 (13.4)	.26
Deep	207 (90.0)	194 (86.6)	
Thalamic ICH location	149 (64.8)	144 (64.3)	.91
Parenchymal ICH volume on admission, mL^d^	8.8 (4.4-15.6)	8.5 (4.4-13.8)	.38
IVH volume on admission, mL^d^	23.4 (13.3-37.1)	23.5 (14.2-38.9)	.64
Parenchymal ICH volume at stability, mL^d^	9.0 (4.2-16.3)	8.8 (3.6-15.3)	.35
IVH volume at stability, mL^d^	20.8 (12.5-33.6)	20.9 (12.4-35.0)	.88
End-of-treatment parenchymal ICH volume, mL^d^	7.3 (2.5-14.4)	8.6 (3.1-14.1)	.32
End-of-treatment IVH volume, mL^d^	5.6 (1.9-13.0)	9.9 (5.6-20.8)	<.001
Withdrawal of care	25 (10.9)	29 (12.9)	.49
EVD ipsilateral to the ICH	133 (57.8)	139 (56.9)	.06
More than 1 EVD	88 (38.2)	95 (42.4)	.58
No. of doses of study agent^d^	12 (9-12)	5 (3-8)	<.001
Time from ictus to first dose of study agent, d^d^	2.4 (1.9-3.0)	2.3 (1.8-2.9)	.12

Abbreviations: EVD, external ventriculostomy drain; ICH, intracerebral hemorrhage; IVH, intraventricular hemorrhage.

^a^ *P* < .05 was considered statistically significant.

^b^ Race and ethnicity were investigator-reported in the CLEAR III trial.

^c^ Other included Asian, Native American, Native Hawaiian, or other Pacific Islander.

^d^ Indicates values presented as median (IQR).

**Figure.**
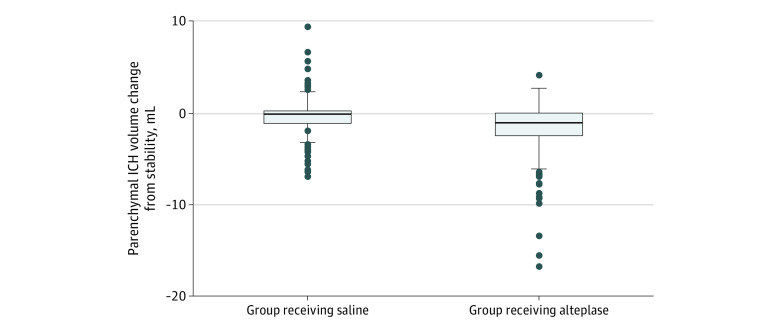
Change in the Parenchymal Intracerebral Hemorrhage (ICH) Volume From Baseline to the End of Treatment, Stratified by Use of Intraventricular Alteplase vs Saline

### Primary Analysis Findings

Results of the bivariate linear regression are shown in eTable 1 in the Supplement. In the unadjusted linear regression analysis (Table 2), alteplase use was associated with a change in parenchymal ICH volume (β, 1.37; 95% CI, 0.92-1.81; *P* < .001). The multivariable linear regression model adjusted for sex, race and ethnicity, stability ICH and IVH volumes, ICH location, and time from ictus to first dose of alteplase (Table 2), showed an association between intraventricular alteplase administration and change in the parenchymal ICH volume (per 1 mL change: β, 1.20; 95% CI, 0.79-1.62; *P* < .001). We performed 2 additional analyses to address heteroscedasticity and found (1) a β of 0.99 (95% CI, 0.64-1.33; *P* < .001) by using robust SEs and (2) a β of 0.78 (95% CI, 0.49-1.06; *P* < .001) by using weighted least squares regression, whereby we weighted for the stability ICH volume and time from ictus to the first dose of alteplase (eFigures 1-3 and eTable 2 in the Supplement).

**Table 2.** Factors Associated With a Change in the Parenchymal Intracerebral Hemorrhage Volume

Model/variable	β (95% CI)	*P* value^a^
Unadjusted analysis		
Alteplase use	1.37 (0.92 to 1.81)	<.001
Multivariable analysis		
Alteplase use	1.20 (0.79 to 1.62)	<.001
Age	0.01 (–0.01 to 0.02)	.58
Female sex^b^	0.63 (0.22 to 1.05)	.003
Black race^c^	0.30 (–0.14 to 0.73)	.19
ICH volume, stability	0.08 (0.06 to 0.11)	<.001
IVH volume, stability	0.02 (0.01 to 0.04)	<.001
Deep ICH location	0.94 (0.27 to 1.61)	.006
Time from ictus to first dose of study agent	–0.49 (–0.78 to –0.20)	.001

Abbreviations: ICH, intracerebral hemorrhage; IVH, intraventricular hemorrhage.

^a^ *P* < .05 was considered to be statistically significant.

^b^ Male sex was the referent.

^c^ Black race was considered separately because Black patients had higher ICH severity and poor outcomes.

### Secondary and Subgroup Analyses Findings

Unadjusted logistic regression analyses showed no association between a change in the parenchymal ICH volume and poor functional outcome (odds ratio [OR], 1.05; 95% CI, 0.94-1.24; *P* = .12) or between a change in parenchymal ICH volume and mortality (OR, 1.04; 95% CI, 0.95-1.15; *P* = .33). In the multivariable logistic regression models, no association was observed between a reduction in ICH volume and poor functional outcome (OR, 0.97; 95% CI, 0.87-1.10; *P* = .64) or mortality (OR, 0.97; 95% CI, 0.99-1.08; *P* = .59) (Table 3).

**Table 3.** Association Between Change in Parenchymal Intracerebral Hemorrhage Volume and Outcomes

Outcome	Modified Rankin Scale score, 4-6 at 6 mo^a^		Mortality at 6 mo^a^	
	OR (95% CI)	*P* value^b^	OR (95% CI)	*P* value^b^
Unadjusted analysis				
Entire cohort (unadjusted)	1.05 (0.94-1.24)	.12	1.04 (0.95-1.15)	.33
Multivariable analysis				
Entire cohort	0.97 (0.87-1.10)	.64	0.97 (0.99-1.08)	.59
Baseline IVH volume				
<20 mL	0.92 (0.78-1.10)	.35	0.82 (0.65-1.02)	.08
≥20 mL	1.11 (0.95-1.30	.18	1.05 (0.93-1.18)	.40
Admission ICH volume				
<10 mL	1.11 (0.92-1.32)	.27	1.02 (0.85-1.21)	.84
≥10 mL	0.96 (0.83-1.12)	.67	1.01 (0.89-1.13)	.90
Thalamic ICH location	0.91 (0.78-1.06)	.23	0.90 (0.79-1.02)	.13
Nonthalamic ICH location	1.05 (0.88-1.25)	.55	1.13 (0.94-1.35)	.19

Abbreviations: ICH, intracerebral hemorrhage; IVH, intraventricular hemorrhage; OR, odds ratio.

^a^ Model was adjusted for age, sex, race and ethnicity, admission IVH volume, parenchymal hematoma location, withdrawal of care, and treatment randomization arm.

^b^ *P* < .05 was considered to be statistically significant.

In the prespecified subgroup analyses, a change in parenchymal ICH volume was not associated with functional outcomes in baseline IVH volume, admission ICH volume, and ICH location (Table 3).

## Discussion

Among patients with a large IVH who were enrolled in the CLEAR III trial, we found that use of intraventricular alteplase was associated with a small reduction in parenchymal ICH volume compared with saline. Although a change in ICH volume was not associated with improved functional outcomes or mortality in the presence of a large IVH, intraventricular alteplase may be a novel approach to facilitating hematoma retraction to improve outcomes in the absence of IVH. More fundamentally, the findings suggest that communication occurs between the intraventricular and intraparenchymal hemorrhage components and that ICH volumes did not increase in response to exposure to intraventricular alteplase.

A recent pilot study suggested a possible benefit of parenchymal ICH volume reduction with intraventricular alteplase use, but the study was limited by a small sample size from a single center, retrospective unblinded design, and measurement of parenchymal ICH volumes without blinding.^9^ In the context of these shortcomings, the current cohort study, which used data from a randomized clinical trial with blinded interventions and end point assessments, has a more robust design for evaluating the potential benefit of intraventricular alteplase for ICH volumes. This study, however, found no association between a change in parenchymal ICH volume and improved outcomes. The mean reduction in ICH parenchymal volume was 1.8 mL in the alteplase group and 0.4 mL in the saline group, which represents a small decrease and therefore did not offer any benefit from a disability standpoint. The pilot study, which had similar initial ICH volumes, showed a similar decrease after the first dose of alteplase and then further substantial reduction after the second and third doses.^9^ Furthermore, differences in the alteplase administration regimen may have also altered the outcomes. The pilot study used 2.5 mg of alteplase starting at 12 hours from the initial CT scan, which was continued every 12 hours until sufficient clearance. As a result, a significant decrease in the parenchymal volume was observed in the first 48 to 72 hours.^9^ The CLEAR III trial, on the other hand, used a lower dose of alteplase, which was started much later after the initial CT scan (mean [SD] of 2.4 [0.5] days).^7^ In addition, we found a significant association between earlier time to first dose of alteplase and greater ICH volume reduction. Thus, these findings support further exploration of earlier administration of intraventricular alteplase to decrease not only the burden of IVH but also ICH volume. Further exploration of the subset of patients with ICH who may benefit from intraventricular thrombolysis, such as those with moderate to large ICH with IVH, especially in thalamic location, may yield useful information.

The findings of this study highlight several points. First, an additive effect of IVH and ICH reduction may be associated with improved functional outcome. Prespecified subgroup analyses in the CLEAR III trial demonstrated lesser disability with the removal of greater than 80% of the initial IVH volume.^7^ However, the mean IVH removal was about 65% in the alteplase group,^7^ suggesting that a residual confounding effect of the remaining IVH may have masked a potential benefit of ICH volume contraction for the outcomes in this study. Second, the passage of alteplase from within the ventricles to the brain parenchyma highlights the intricate communications between different compartments of the brain. Emerging studies indicate the existence of a so-called glymphatic system, wherein the cerebrospinal fluid flows into the perivascular space around cerebral arteries, combining with interstitial fluid and parenchymal solutes, and exiting down venous perivascular spaces.^14,15^ This pathway represents a para-arterial influx route for the cerebrospinal fluid to enter the brain parenchyma, a route that could serve as a conduit for the flow of alteplase from the ventricular system to the brain parenchyma.

### Limitations

This study has some limitations. First, the strict inclusion criteria of the CLEAR III trial, along with the inclusion of small parenchymal ICH volumes, limit the generalizability of the results, but the blinded intervention and outcome assessment minimize any other potential biases. Second, because this study was a post hoc exploratory analysis, its findings might represent a statistical artifact by being among the multiple hypotheses tested in the same data set. However, given that a previous pilot study found similar results suggests that the association between alteplase use and parenchymal ICH volume contraction is a reproducible finding that can inform the design of future trials. Third, changes in the IVH volume may have resulted in misclassification of parenchymal ICH volumes. Although semiautomated technology was used to ascertain ICH and IVH volumes, the demarcation of the hematoma and IVH volumes may not be completely accurate, particularly in the case of deep hemorrhages. Fourth, this study included few patients with anticoagulation-related ICH (n = 40), which precluded any meaningful analyses. In addition, coagulopathy had to be corrected and ICH and IVH stability had to be demonstrated before the study intervention could be initiated. As a result, we were unable to study the role of alteplase in anticoagulant-associated ICH. Fifth, the CLEAR III trial population had small ICH volumes (<30 mL) for whom a 1- to 2-mL mean reduction was likely not sufficient to be associated with functional improvement. Whether the benefit of alteplase is greater in patients with larger ICH volumes is unclear. Sixth, we did not observe a significant association of ICH volume contraction with clinical outcome and thus did not show an immediate clinical benefit. Given that numerous ICH studies have found an association between parenchymal ICH volume and poor outcome,^16,17^ an overwhelmingly large IVH burden may have masked the potential role of alteplase in ICH volume contraction. Furthermore, this study likely lacked the power to detect an association between a change in the ICH volume and functional outcomes. To demonstrate a clinical benefit, future trials may have to carefully select and prospectively assess patients for ICH volume changes while undergoing intraventricular thrombolysis.

## Conclusions

This cohort study found an association between intraventricular alteplase administration and small reductions in parenchymal ICH volume. However, no association was observed between change in ICH volume and improved functional outcomes or mortality. Examining patients with ICH and IVH who may benefit from intraventricular thrombolysis, such as those with moderate to large ICH in thalamic location, may yield useful information.

## Notes

Supplement. **eTable 1.** Factors Associated With a Change in the Parenchymal ICH Volume in Simple Linear Regression Models **eFigure 1.** Standardized Probability Plot for the Linear Regression Model **eFigure 2.** Kernel Density Estimate for the Linear Regression Model **eFigure 3.** Residual vs Fitted Plot for the Linear Regression Model **eTable 2.** Multiple Linear Regression Analysis Showing the Factors Associated With a Change in the Parenchymal ICH Volume
