# Supplementary material for: Association Between Intraventricular Alteplase Use and Parenchymal Hematoma Volume in Patients With Spontaneous Intracerebral Hemorrhage and Intraventricular Hemorrhage
Source: JAMA Netw Open. 2021 Dec 3;4(12):e2135773. doi: 10.1001/jamanetworkopen.2021.35773 (PMC8642781; doi:10.1001/jamanetworkopen.2021.35773)
Supplement: Supplement. — eTable 1. Factors Associated With a Change in the Parenchymal ICH Volume in Simple Linear Regression Models eFigure 1. Standardized Probability Plot for the Linear Regression Model eFigure 2. Kernel Density Estimate for the Linear Regression Model eFigure 3. Residual vs Fitted Plot for the Linear Regression Model eTable 2. Multiple Linear Regression Analysis Showing the Factors Associated With a Change in the Parenchymal ICH Volume [file jamanetwopen-e2135773-s001.pdf]

Supplementary Online Content

Witsch J, Roh DJ, Avadhani R, et al. Association between intraventricular alteplase use and parenchymal hematoma volume in patients with spontaneous intracerebral hemorrhage and intraventricular hemorrhage. *JAMA Netw Open*. 2021;4(12):e2135773. doi:10.1001/jamanetworkopen.2021.35773

eTable 1. Factors Associated With a Change in the Parenchymal ICH Volume in Simple Linear Regression Models

eFigure 1. Standardized Probability Plot for the Linear Regression Model

eFigure 2. Kernel Density Estimate for the Linear Regression Model

eFigure 3. Residual vs Fitted Plot for the Linear Regression Model

eTable 2. Multiple Linear Regression Analysis Showing the Factors Associated With a Change in the Parenchymal ICH Volume

This supplementary material has been provided by the authors to give readers additional information about their work.

© 2021 Witsch J et al. *JAMA Network Open.*

eTable 1. Factors Associated With a Change in the Parenchymal ICH Volume in Simple Linear Regression Models

Covariates	Univariable Analysis	
	Beta (95% CI)	P value
Age	0.01 (-0.01 to 0.02)	0.58
Female gender	0.46 (-0.01 to 0.91)	0.05
Black race	0.17 (-0.11 to 0.65)	0.09
Hypertension	0.08 (-0.43 to 0.60)	0.76
Hyperlipidemia	0.37 (-0.95 to 1.70)	0.58
Tobacco use	0.07 (-0.46 to 0.59)	0.81
Prior anticoagulant use	-0.35 (-1.16 to 0.46)	0.39
Prior antiplatelet use	0.22 (-0.30 to 0.75)	0.40
Glasgow Coma Scale at screening	-0.15 (-0.22 to -0.09)	<0.001
ICH location<br>Deep (vs. lobar)	1.44 (0.75 to 2.15)	<0.001
Thalamic ICH location	0.55 (0.07 to 1.03)	0.02
ICH volume on admission	0.09 (0.06 to 0.12)	<0.001
IVH volume on admission	0.01 (-0.01 to 0.03)	0.46
ICH volume at stability	0.09 (0.06 to 0.12)	<0.001
IVH volume at stability	0.01 (0.01 to 0.04)	0.03
End of treatment ICH volume	-0.01 (-0.03 to 0.03)	0.71
End of treatment IVH volume	-0.02 (-0.03 to -0.01)	0.02
EVD ipsilateral to the ICH	0.07 (-0.28 to 0.18)	0.78
More than 1 EVD	0.14 (-0.12 to 0.26)	0.67
Alteplase	1.37 (0.92-1.81)	<0.001
Number of doses of study agent	0.02 (-0.04 to 0.07)	0.54
Time from ictus to first dose of study agent	-0.67 (-0.98 to -0.36)	<0.001

Abbreviations: CI: Confidence interval; dL: deciliter, CSF: Cerebrospinal fluid; EVD: External ventriculostomy drain; ICH: Intracerebral hemorrhage; ICP: Intracranial pressure; IVH: Intraventricular hemorrhage, mg: milligrams, mL: milliliters; OR: Odds ratio, RBC: red blood cell; WBC: white blood cell.
P value <0.05 was considered significant

© 2021 Witsch J et al. *JAMA Network Open.*

eFigure 1. Standardized Probability Plot for the Linear Regression Model

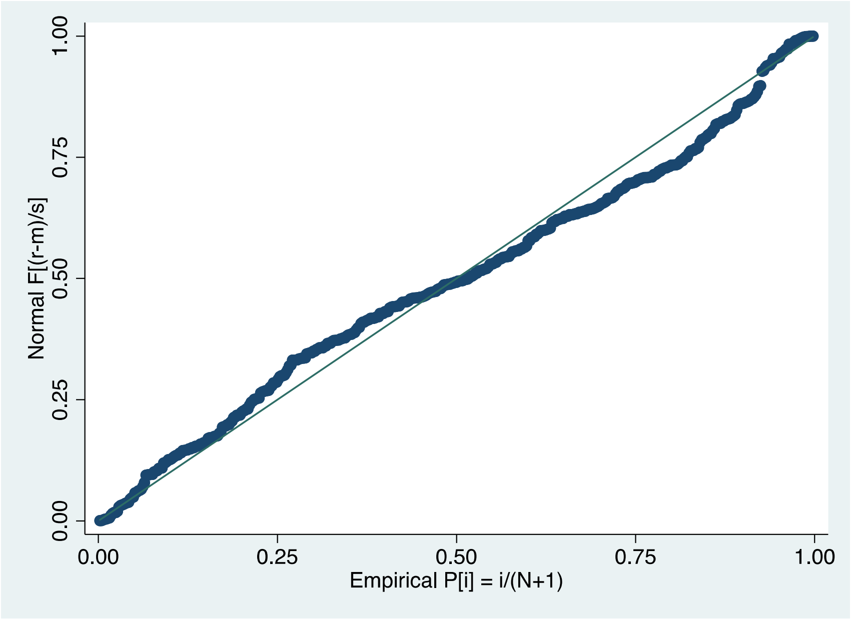

© 2021 Witsch J et al. *JAMA Network Open.*

eFigure 2. Kernel Density Estimate for the Linear Regression Model

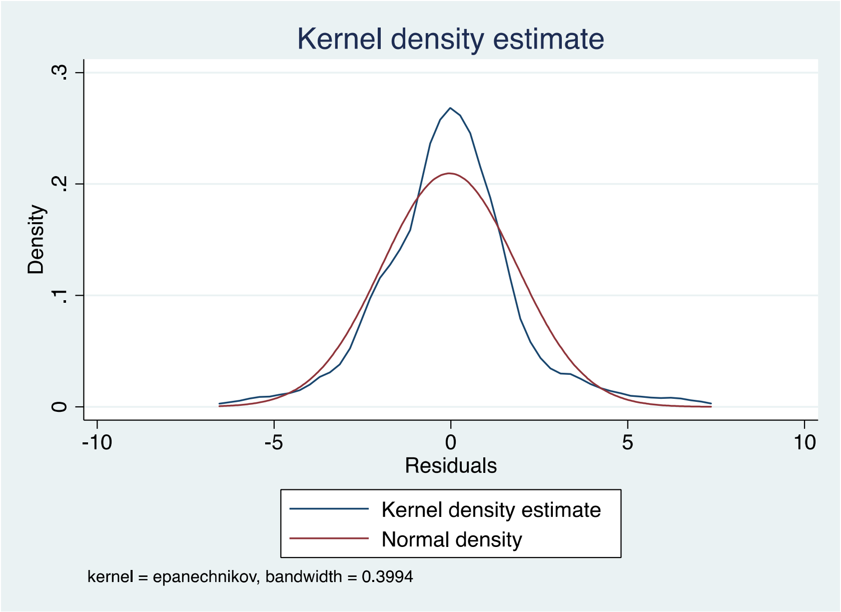

© 2021 Witsch J et al. *JAMA Network Open.*

eFigure 3. Residual vs Fitted Plot for the Linear Regression Model

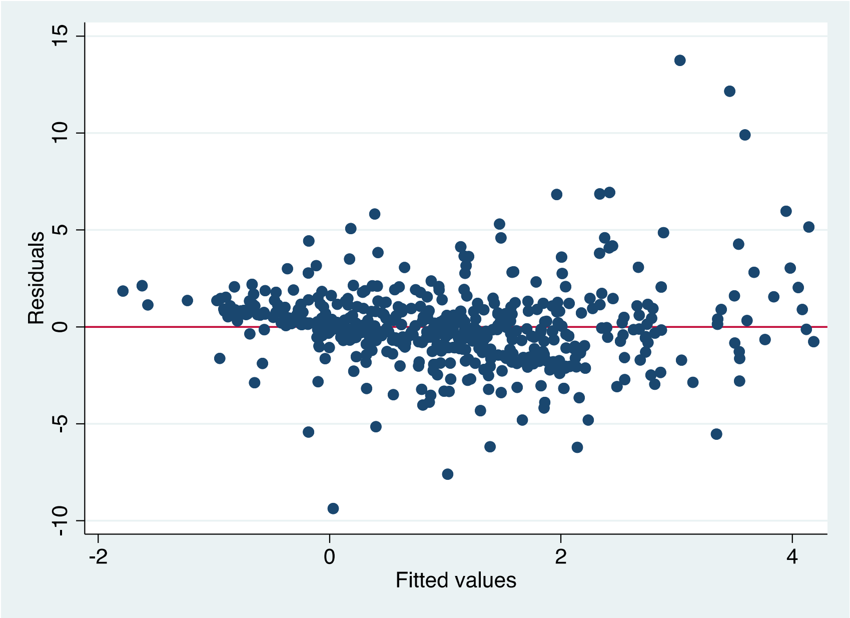

© 2021 Witsch J et al. *JAMA Network Open.*

eTable 2. Multiple Linear Regression Analysis Showing the Factors Associated With a Change in the Parenchymal ICH Volume

	Robust Standard Errors		Weighted Least Squares Regression	
	**Beta (95% CI)**	**P value**	**Beta (95% CI)**	**P value**
Alteplase use	0.99 (0.64-1.34)	<0.001	0.78 (0.49 to1.06)	<0.001
Age	0.01 (-0.01 to 0.04)	0.85	0.02 (-0.015 to 0.05)	0.73
Female sex	0.56 (0.19 to 0.93)	0.003	0.33 (0.05 to 0.61)	0.02
Black race	0.23 (-0.16 to 0.64)	0.23	0.13 (-0.17 to 0.45)	0.39
ICH volume, stability	0.07 (0.04 to 0.10)	<0.001	0.09 (0.06 to 0.12)	<0.001
IVH volume, stability	0.01 (0.001 to 0.02)	0.03	0.01 (-0.01 to 0.05)	0.12
Deep ICH location	0.67 (0.15-1.18)	0.01	0.49 (0.11 to 0.88)	0.01
Time from ictus to first dose of study agent	-0.52 (-0.78 to -0.26)	<0.001	-0.34 (-0.54 to -0.13)	0.001

Abbreviations: CI, confidence interval; ICH, intracerebral hemorrhage; IVH, intraventricular hemorrhage.
P value <0.05 was considered significant

© 2021 Witsch J et al. *JAMA Network Open.*
